# *Artemisia capillaris* inhibits atopic dermatitis-like skin lesions in *Dermatophagoides farinae*-sensitized Nc/Nga mice

**DOI:** 10.1186/1472-6882-14-100

**Published:** 2014-03-14

**Authors:** Hyekyung Ha, Hoyoung Lee, Chang Seob Seo, Hye-Sun Lim, Jun Kyoung Lee, Mee-Young Lee, Hyeunkyoo Shin

**Affiliations:** 1 Herbal Medicine Formulation Research Group, Korea Institute of Oriental Medicine, 1672 Yuseongdae-ro, Daejeon, Yuseong-gu 305-811, Republic of Korea; 2 KM Health Technology Research Group, Korea Institute of Oriental Medicine, 1672 Yuseongdae-ro, Daejeon, Yuseong-gu 305-811, Republic of Korea

**Keywords:** *Artemisia capillaries* Thunb., Atopic dermatitis, Anti-inflammation, HPLC

## Abstract

**Background:**

*Artemisia capillaries* Thunb. (AC) has been used to treat inflammatory and hepatic disorders such as hepatic injury, hepatic fibrosis and hepatitis. However, the efficacy of AC against atopic dermatitis (AD), an inflammatory disease, has not been examined. In the present study, AC was evaluated for anti-inflammatory and anti-AD effects using both *in vitro* and *in vivo* systems.

**Methods:**

The contents of six compounds (chlorogenic acid, caffeic acid, isochlorogenic acid A, hyperoside, isoquercitrin and scoparone) in AC were simultaneously assayed using HPLC system. To evaluate the anti-inflammatory effect of AC, NO production was measured in RAW264.7 cell stimulated with 1 μg/mL LPS. Histamine levels were assayed in MC/9 cells stimulated with 50 nM PMA and 1 μM A23187. To examine the role of AC *in vivo*, AC (10 mg/mouse/day) was topically applied for four weeks the back and ears of *Dermatophagoides farinae*-sensitized Nc/Nga mice. Protopic ointment (0.1% tacrolimus) was used as a positive control.

**Results:**

The contents of the six components in AC range from 0.44 to 43.14 mg/g. Chlorogenic acid (21.06 ± 0.08 mg/g) and isochlorogenic acid A (43.14 ± 0.12 mg/g) were major components in AC. AC inhibited NO and histamine production in cells respectively. In *D. farinae*-sensitized Nc/Nga mice, the topical application of AC reduced dermatitis scores, hemorrhage, hypertrophy and hyperkeratosis of the epidermis in the dorsal skin and ear. The treatment of AC also reduced the plasma levels of histamine (1.5 fold) and IgE (1.4 fold).

**Conclusions:**

Our results suggest that AC should be explored as a potential therapeutic agent to treat atopic dermatitis and analysis by HPLC will help to improve the quality of AC.

## Background

Atopic dermatitis (AD) is an inflammatory, chronically relapsing, non-contagious and pruritic skin disorder [1]. AD is often accompanied by allergic inflammation, which is initiated by activation of the adaptive immune response. Immunoglobulin E (IgE) is produced in plasma cells and bound by mast cells in type I allergic reactions. The IgE-primed mast cells release chemical mediators, such as histamine, leukotrienes (LTs) and prostaglandin D_2_ (PGD_2_). These mediators lead to immediate phase reactions in the tissue, such as redness and itching, shortly after allergen-IgE binding. In the later phases of the disease, cytokines (IL-4 and IL-13) and chemokines are generated and released several hours after allergen-antibody cross-linking [2].

Topical corticosteroids are currently the most potent treatment for AD. However, patients with more severe forms of the disease do not always respond satisfactorily to these agents. Chronic use can also be associated with significant adverse effects. The long-term use of corticosteroids results in tachyphylaxis and treatment resistance. Therefore, it would be advantageous to develop new treatments that lack the side effects of corticosteroids [3]. The use of systemic corticosteroids is known to be effective in the short-term treatment of AD. However, no studies exist to support their long-term use, and both rebound flaring and long-term side effects are limiting factors [4]. Immunosuppressive drugs, including calcineurin inhibitors such as cyclosporine, tacrolimus and pimecrolimus, have been reported to be effective for atopic dermatitis. However, concerns over systemic toxicity have limited their use [5,6]. Tacrolimus has been developed for the treatment of moderate to severe AD, but topical tacrolimus ointment causes transient burning in ~60% of patients [6]. Consequently, the need to efficiently manage the AD response while reducing side effects has led to the development of alternative remedies.

*Artemisia capillaris* Thunb. (AC) has been traditionally used as an herbal medicine to treat pyrexia and liver disorders in East Asia. Several studies have also established that AC inhibits chemical-induced oxidative stress, hepatic injury, hepatic fibrosis, hepatitis and obesity [7-10]. Additionally, Kim et al. [11] reported that AC extracted with boiling water inhibits cytokine-induced nitric oxide (NO) formation in a rat insulinoma cell line. However, the efficacy of AC in treating AD has not been examined.

In the present study, we evaluated the anti-inflammatory and anti-allergic effects of AC by measuring its inhibition of NO production in lipopolysaccharide (LPS)-treated RAW264.7 cells. Furthermore, we analyzed histamine production in MC/9 cells stimulated with phorbol-12 myristate 13-acetate (PMA) and A23187, in addition to analyzing the AD response in Nc/Nga mice.

## Methods

### Plant materials and extract

*A. capillaris* was purchased from Kwangmyungdang Medicinal herbs (Ulsan, Korea) in September 2009. These materials were confirmed taxonomically by Professor Je-Hyun Lee of Dongguk University, Korea. A voucher specimen (AC-2009-EBM30) has been deposited at the Herbal Medicine Formulation Research Group at the Korea Institute of Oriental Medicine.

The 300 g sample of dried *A. capillaris* was extracted with 70% EtOH (3 L × 3) by sonication for 60 min. The extract solution was filtered through Whatman No. 2 filter paper (150 mm diameter, Buckinghamshire, UK) and evaporated to dryness using a rotary evaporator. The yield of 70% EtOH extract was 8.30% (24.89 g).

### Chemicals and reagents

Chlorogenic acid and caffeic acid were purchased from Acros Organics (Pittsburgh, PA, USA). Hyperoside and scoparone were purchased from Sigma-Aldrich (St. Louis, MO, USA). Isoquercitrin and isochlorogenic acid A were purchased from Biopurify Phytochemicals Ltd. (Chengdu, China). The purity of the six compounds was determined to be ≥97% by HPLC analysis. HPLC-grade reagents, methanol, acetonitrile, and water were obtained from J.T.Baker (Phillipsburg, NJ, USA). Glacial acetic acid was of analytical reagent grade and was procured from Junsei (Tokyo, Japan).

### Chromatographic conditions of HPLC analysis

The HPLC analysis was performed using a Shimadzu LC-20A HPLC system (Shimadzu Co., Kyoto, Japan) consisting of a solvent delivery unit, an on-line degasser, a column oven, an autosampler and a PDA detector. The data processor used LC Solution software (version 1.24, Shimadzu Co., Kyoto, Japan). The analytical column used was a Gemini C_18_ (250 × 4.6 mm; particle size 5 μm; Phenomenex, Torrance, CA, USA) maintained at 40°C. The mobile phases were composed of 1.0% (v/v) aqueous acetic acid (A) and 1.0% (v/v) acetic acid in acetonitrile (B). The gradient flow rate was as follows: 0–5 min, 0–10% B; 5–30 min, 10–50% B; 30–35 min, 50–50% B; 35–40 min, 50–10% B. The flow rate and injection volume were 1.0 mL/min and 10 μL, respectively. The detection wavelength was set at 254 nm for hyperoside and isoquercitrin, at 320 nm for chlorogenic acid, caffeic acid, and isochlorogenic acid A and at 340 nm for scoparone.

### Preparations of standard and sample solutions

**Figure 1:**
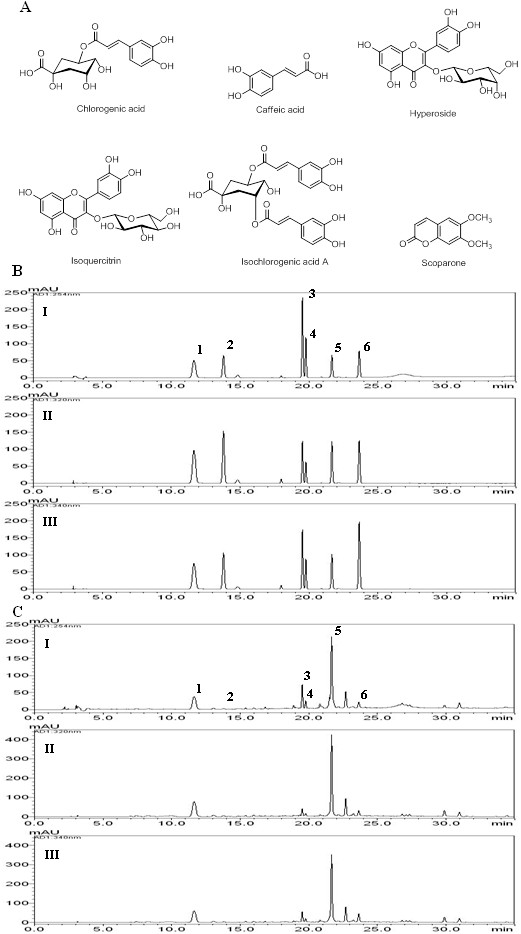
**Chemical structures and HPLC chromatograms. A**: Chemical structures of six marker components of A. capillaris. **B**: Representative HPLC chromatogram of reference standards. **C**: Representative HPLC chromatogram of A. capillaris extract. The detection wavelength was set at 254 nm (I) for hyperoside (3) and isoquercitrin (4), at 320 nm (II) for chlorogenic acid (1), caffeic acid (2), and isochlorogenic acid A (5) and at 340 nm (III) for scoparone (6).

Standard stock solutions of three phenolic acids (chlorogenic acid, caffeic acid and isochlorogenic acid A), two flavonoids (hyperoside and isoquercitrin) and one coumarin (scoparone) (Figure 1A) were dissolved in methanol at concentrations of 1.0 mg/mL and kept at 4°C. The working standard solutions were diluted to calibration curves in the concentration range of 0.78–50.00 μg/mL for chlorogenic acid, hyperoside and scoparone, 0.16–10.00 μg/mL for caffeic acid and isoquercitrin and 2.34–150.00 μg/mL for isochlorogenic acid A.

The 70% EtOH extract (20 mg) was dissolved in 70% EtOH (10 mL) and then filtered through a 0.2 μm membrane filter (Woongki Science, Seoul, Korea) before injection into the HPLC for simultaneous analysis.

### Measurement of nitric oxide (NO) production in LPS-treated RAW264.7 cells

RAW 264.7 murine macrophage cells were obtained from the American Type Culture Collection (ATCC, Rockville, MD, U.S.A.) and maintained in Dulbecco’s modified Eagle’s medium (DMEM, Gibco BRL., NY, U.S.A.) supplemented with 5.5% (v/v) fetal bovine serum (FBS, Gibco BRL., NY, U.S.A.), 100 U/mL penicillin and 100 μg/mL streptomycin (Gibco BRL, NY, U.S.A.). The cells were seeded at densities of 2.5×10^3^ cells/well in 96-well plates for the cytotoxicity assay. The cells were then incubated with different concentrations of herbal extracts (10, 50 and 100 μg/mL) for 24 hr. The vehicle control was 1% DMSO. After treatment, 10 μL of Cell Counting Kit-8 reagent (CCK-8, Dojindo, Japan) was added to each well and the plates were incubated for 4 hr. The absorbance was measured at 450 nm using a microplate reader (BenchmarkPlus, Bio-Rad Laboratories Inc., U.S.A.) and the percentages of viable cells were calculated. AC extracts in the range of 20–200 μg/mL did not cause cytotoxicity in RAW 264.7 cells and non-cytotoxic concentrations of herbal extracts were used for the subsequent experiments.

RAW 264.7 cells were seeded at a density of 5×10^5^ cells in a 48-well plate for the NO assay. After culturing for 16 hr, the cells were stimulated with 1 μg/ml of LPS in the presence or absence of AC extracts (20–200 μg/mL) for 18 hr. *N*^*G*^-methyl-L-arginine (NMMA; Sigma-Aldrich, Inc., MO, U.S.A.) and indomethacin (Sigma-Aldrich, Inc., MO, U.S.A.) were used as positive controls. A Griess reagent system (Promega., WI, U.S.A.) was used to measure the production of NO in the culture supernatants. Briefly, samples (50 μL/well) were incubated at room temperature with 1% sulfanilamide for 10 min and with 1% α-naphthylamine for 10 min. The absorbance was then evaluated at 535 nm using a calibration curve generated using the standards.

### Measurement of histamine in PMA/A23187-treated MC/9 cells

The murine mast cell line MC/9 was maintained in DMEM media containing 10% (v/v) FBS, 0.05 mM 2-mercaptoethanol (Sigma Chemical Co., MO, U.S.A.), 10% (v/v) Rat T-STIM (BD Biosciences, MA, U.S.A.), 100 U/mL of penicillin and 100 μg/mL of streptomycin in a humidified 5% CO_2_ atmosphere. The MC/9 cells were plated in 48-well plates at a concentration of 2×10^5^ cells per well. The cells were either untreated or treated with either phorbol 12-myristate 13-acetate (50 nM; PMA, Sigma-Aldrich, Inc., MO, U.S.A.) and A23187 (1 μM; Sigma-Aldrich, Inc., MO, U.S.A.) alone or PMA/A23187 (PA) + AC (50–200 μg/mL) for 24 hr. The histamine levels in the MC/9 cell supernatants were measured by ELISA in accordance with the manufacturer’s instructions (Oxford Biomedical Research, U.S.A.).

### Animals and sensitization

Male Nc/Nga mice (8 weeks old) were obtained from Central Laboratory Animal Inc. (Seoul, Korea) and housed individually in an air-conditioned room maintained at 24 ± 2°C with 55 ± 15% relative humidity. The animals were allowed to acclimatize for 2 weeks before the experiments were initiated. All experimental procedures were carried out in accordance with the NIH Guidelines for the Care and Use of Laboratory Animals and were approved by Korea Institute of Oriental Medicine Institutional Animal Care and Use Committee. The approval number for the animal study was #10-052. The animals were cared for in accordance with the dictates of the National Animal Welfare Law of Korea.

AD-like skin lesions were induced in male Nc/Nga mice using *Dermatophagoides farinae* extract ointment (Biostir-AD, Biostir Co., Ltd., Kobe, Japan) [12]. At ten weeks of age the mice were grouped randomly into four groups with seven mice per group. The mice were divided into untreated (normal; 200 μL of 70% EtOH/mouse/day), *D. farinae*-sensitized (control; 70% EtOH), *D. farinae*-sensitized plus Protopic® ointment-treated (Protopic; 50 mg/mouse/day) and *D. farinae*-sensitized plus AC extract-treated (AC; 10 mg/mouse/day) groups. For sensitization, 50 mg Biostir-AD was topically applied on the upper dorsal skin and the back of the ears twice weekly for 4 weeks. The AC extract was dissolved 70% ethanol and applied every day for 4 weeks.

### Dermatitis score

The dermatitis scores were assessed by evaluating the dorsal skin and the ears once a week for 4 weeks. The Eczema Area and Severity Index (EASI) scoring system was employed to evaluate the severity of dermatitis. The dermatitis score was defined as the sum of the scores for erythema/hemorrhage, edema, excoriation/erosion and scaling/dryness and scored as follows: no symptoms, 0; mild, 1; moderate, 2; and severe, 3 [4].

### Histological analysis

Mice were anesthetized by pentobarbital sodium (Entobar inj., Hanlim Pharm. Co., Ltd., Korea) injection (i.p.). Blood samples were taken and the animals were sacrificed by exsanguination from the aorta. A complete gross observation was performed on all terminated animals. The blood samples were collected in a microtainer (Becton, Dickinson and Company, NJ, USA) containing K_2_-EDTA. Plasma samples were collected after centrifugation at 10000 rpm and stored at -80°C until they were further assayed. The dorsal skin and one ear of each mouse were removed and fixed in 10% (v/v) natural buffered formalin for 24 hr. The tissues were embedded in paraffin and then sectioned at 4 μm thickness. The tissue sections were then stained with hematoxylin & eosin (H&E) or toluidine blue to estimate epidermal inflammation (hypertrophy and infiltration by inflammatory cells) and mast cell counts, respectively. The dermal mast cell content was quantified by counting the numbers of toluidine-blue positive cells in randomly selected high power fields for each specimen.

### Plasma levels of IgE and histamine

The plasma levels of IgE (Bethyl Laboratories Inc., U.S.A.) and histamine (Oxford Biomedical Research, U.S.A.) were measured by ELISA accordance with the manufacturer’s instructions.

### Statistical analysis

The data are expressed as the mean ± SEM and were analyzed using a one-way ANOVA followed by the Bonferroni multiple comparison test. A *P*-value <0.05 was defined as statistically significant. All statistical analyses were performed using the SYSTAT® 8.0 program (SYSTAT Inc., Evanston, IL, U.S.A.).

## Results

### HPLC analyses

The HPLC-PDA method was utilized for simultaneous determination of the chlorogenic acid, caffeic acid, hyperoside, isoquercitrin, isochlorogenic acid A and scoparone content of *A. capillaris* using mobile phases comprised of 1.0% (v/v) acetic acid in water (A) and 1.0% (v/v) acetic acid in acetonitrile (B). Using optimized chromatography conditions, the six compounds were eluted within 30 min in the sample analysis. The correlation coefficients (*r*^2^) of the calibration curves for six components were ≥ 0.9999. The ranges of LOD and LOQ were 38.40–126.49 ng/mL and 128.00–421.62 ng/mL, respectively. The linear equations and the *r*^2^ of the calibration curves are summarized in Table 1. The reproducibility for all analytes showed an RSD of less than 1.0% for peak responses and less than 0.2% for retention times (data not shown). The retention times of chlorogenic acid, caffeic acid, hyperoside, isoquercitrin, isochlorogenic acid A, and scoparone were 11.63, 14.80, 19.53, 19.78, 21.63, and 23.66, respectively. The HPLC chromatograms of the standard solution and water extract of *A. capillaries* are shown in Figure 1B and C. The contents of the six constituents ranged from 0.44–43.14 mg/g. Chlorogenic acid and isochlorogenic acid A were the major components in *A. capillaris* extract (Table 2).

**Table 1:** **Calibration curves for the six marker components of *A. capillaris *** **(n = 3)**

**Component**	**Linear range (μg/mL)**	**Regression equation**^***a***^	***r***^**2**^	**LOD (ng/mL)**	**LOQ (ng/mL)**
Chlorogenic acid	0.78–50.00	Y = 32627.70x – 4523.06	1.0000	126.49	421.62
Caffeic acid	0.16–10.00	Y = 60172.54x – 1297.07	0.9999	43.64	145.45
Hyperoside	0.78–50.00	Y = 25100.81x + 2699.33	0.9999	39.56	132.88
Isoquercitrin	0.16–10.00	Y = 26489.61x + 23.88	1.0000	38.40	128.00
Isochlorogenic acid A	2.34–150.00	Y = 38075.16x – 21283.50	0.9999	51.62	172.06
Scoparone	0.78–50.00	Y = 37297.38x + 3577.02	0.9999	51.24	170.79

^a^Y represents peak area (mAU); x represents concentration (μg/mL).

**Table 2:** **Contents of the six marker compounds in the 70**% **ethanol extract of *A. capillaris *(n = 3)**

**Compound**	**Content (mg/g)**		
	**Mean**	**SD**	**RSD (%)**
Chlorogenic acid	21.06	0.08	0.36
Caffeic acid	0.44	0.01	1.54
Hyperoside	8.44	0.03	0.38
Isoquercitrin	2.96	0.01	0.21
Isochlorogenic acid A	43.14	0.12	0.27
Scoparone	5.56	0.02	0.43

### Inhibitory effects of AC on NO and histamine production

**Figure 2:**
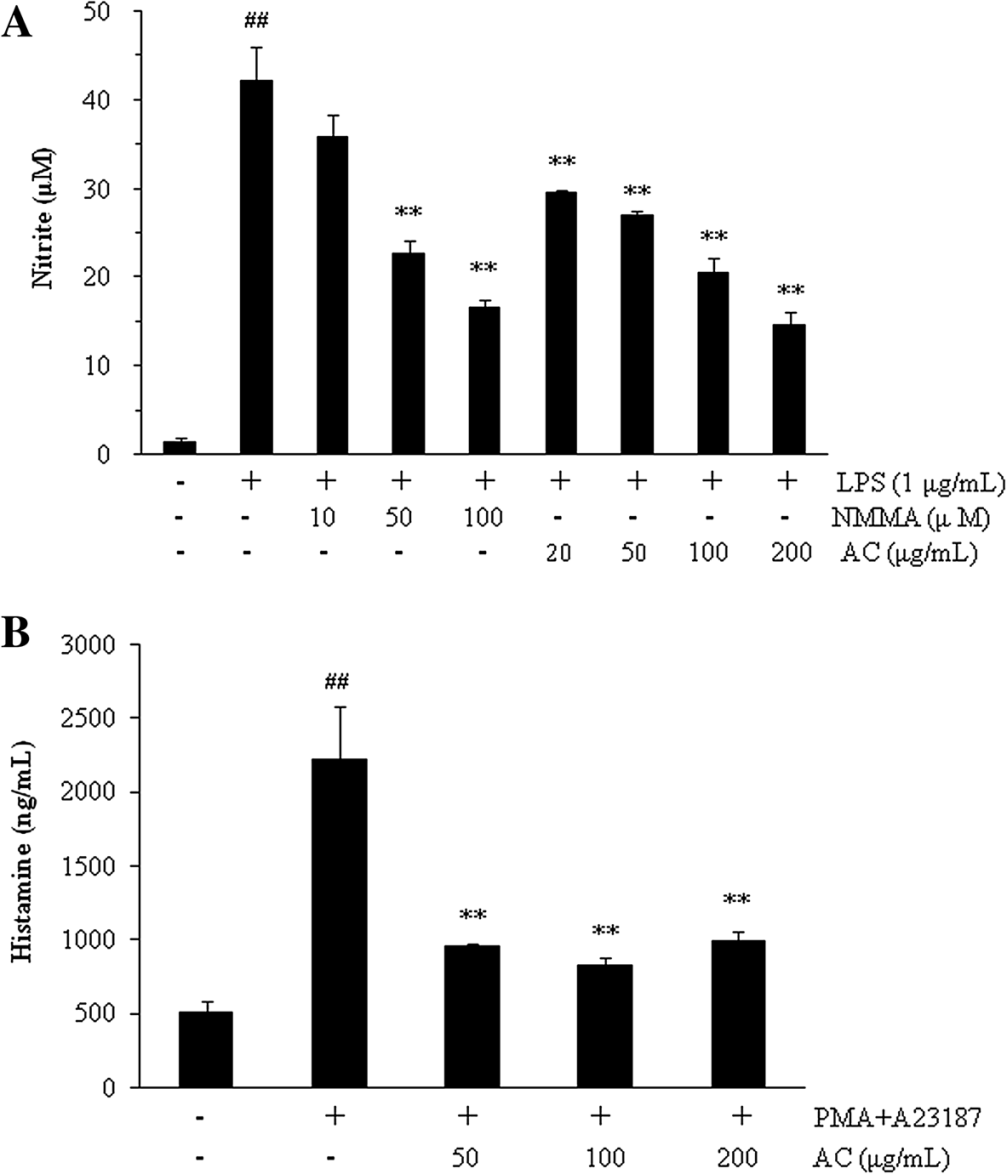
**Inhibitory effects of NO and histamine production by *A. capillaris *extract (AC). A**: AC inhibited NO production in LPS-stimulated (1 μg/mL, 24 hr) RAW 264.7 cells in a concentration-dependent manner (mean ± SEM (*n =* 3), ^##^: *P <* 0*.*01 compared with the control group, **: *P <* 0*.*01 compared with the LPS-treated group). **B**: Histamine production was reduced by AC in PMA and A23187 (PA)-treated (50 nM and 1 μM, 24 hr) MC/9 cells (mean ± SEM (*n =* 3), ^##^: *P <* 0*.*01 compared with the control group, **: *P <* 0*.*01 compared with the PA-treated group).

AC was shown to inhibit the inflammatory response in LPS-stimulated RAW264.7 cells. LPS-induced NO production was significantly reduced by AC extract in a dose-dependent manner compared to the group treated with LPS alone (*P* < 0.01, IC_50_ = 87.82 μg/mL, Figure 2A). The PA-stimulated MC/9 cells treated with AC showed a decline in histamine production compared with that of cells stimulated with PA alone (*P* < 0.01, Figure 2B).

### Dermatitis scores and histological observations in Nc/Nga mice

**Figure 3:**
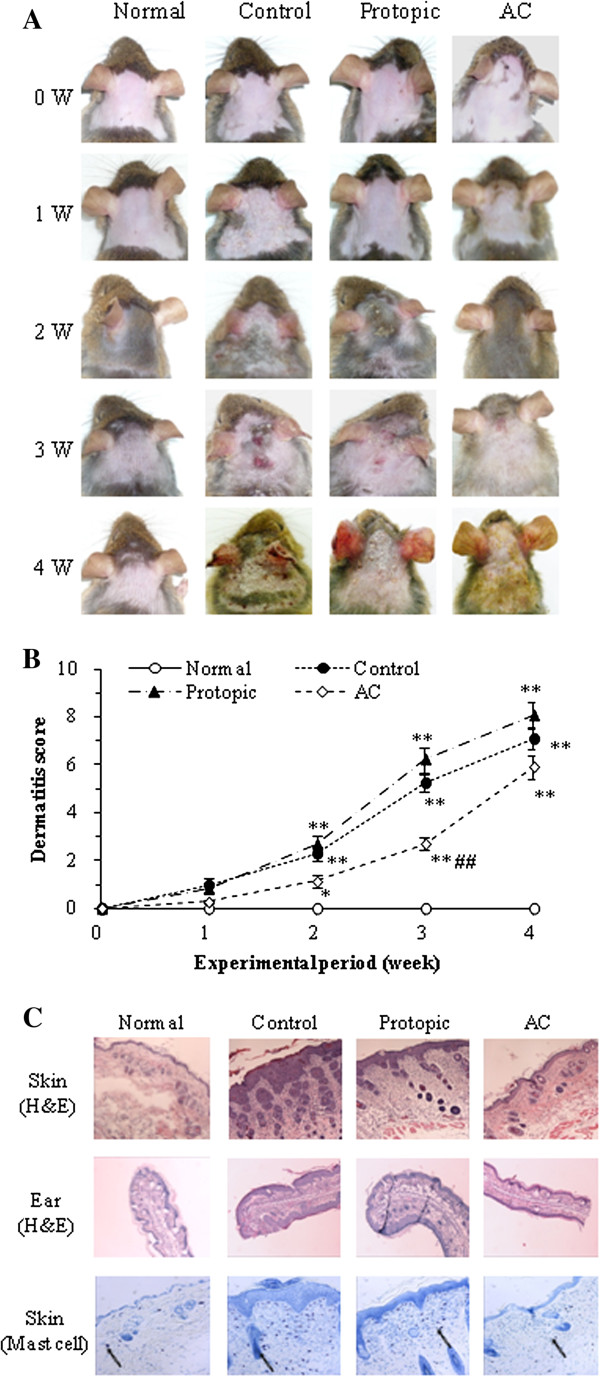
**Dermatitis scores and histological changes in *D. farinae *extract-sensitized Nc/Nga mice. A**: Macroscopic changes following consecutive administration of *A. capillaris* extract (AC) or Protopic ointment to *D. farinae*-induced AD like lesions on the back and ears in Nc/Nga mice. The images show the back and ears 4 weeks after sensitization. **B**: The dermatitis scores of *D. farinae*-induced AD-like lesions on the back and ears (mean *±* SEM (*n =* 5), *: *P <* 0*.*05 and **: *P <* 0*.*01, compared with the normal group, ^##^: *P <* 0*.*01, compared with the *D. farinae*-induced control group). AC (10 mg/mouse/day) and Protopic ointment (50 mg/mouse/day) were topically applied on the back and ears once daily for 4 weeks. **C**: Histological features of the back and ears. The tissues were stained with hematoxylin & eosin (H&E) or toluidine blue to estimate epidermal inflammation or mast cell infiltration. The mast cells are indicated by arrows.

Representative photographs for each group of animals are shown in Figure 3A. Macroscopically, the mice developed lesions on the dorsal skin and ears starting at the second week after the initiation of *D. farinae* extract treatment. In the AC-treated group, the dorsal skin and ear lesion severity was significantly reduced compared to the control group at the third week (*P* < 0.01). However, the dermatitis score of Protopic-treated positive control group was no different than that of the control group (Figure 3B). The maximum dermatitis score was recorded following the fourth week of *D. farinae* extract application in all groups. The scores for each group were as follows: 0.0 ± 0.00 (normal), 7.1 ± 0.43 (control), 8.1 ± 0.54 (Protopic) and 5.9 ± 0.49 (AC) (Figure 3B).

The control and Protopic treatment groups showed significant inflammatory changes, including ear lesions and hemorrhage, hypertrophy, and hyperkeratosis of the epidermis in the dorsal. These changes were significantly ameliorated in the AC treatment group. The infiltration of mast cells in the dorsal skin was reduced by the application of AC to AD-induced mice (Figure 3C).

### Plasma levels of histamine and IgE

**Figure 4:**
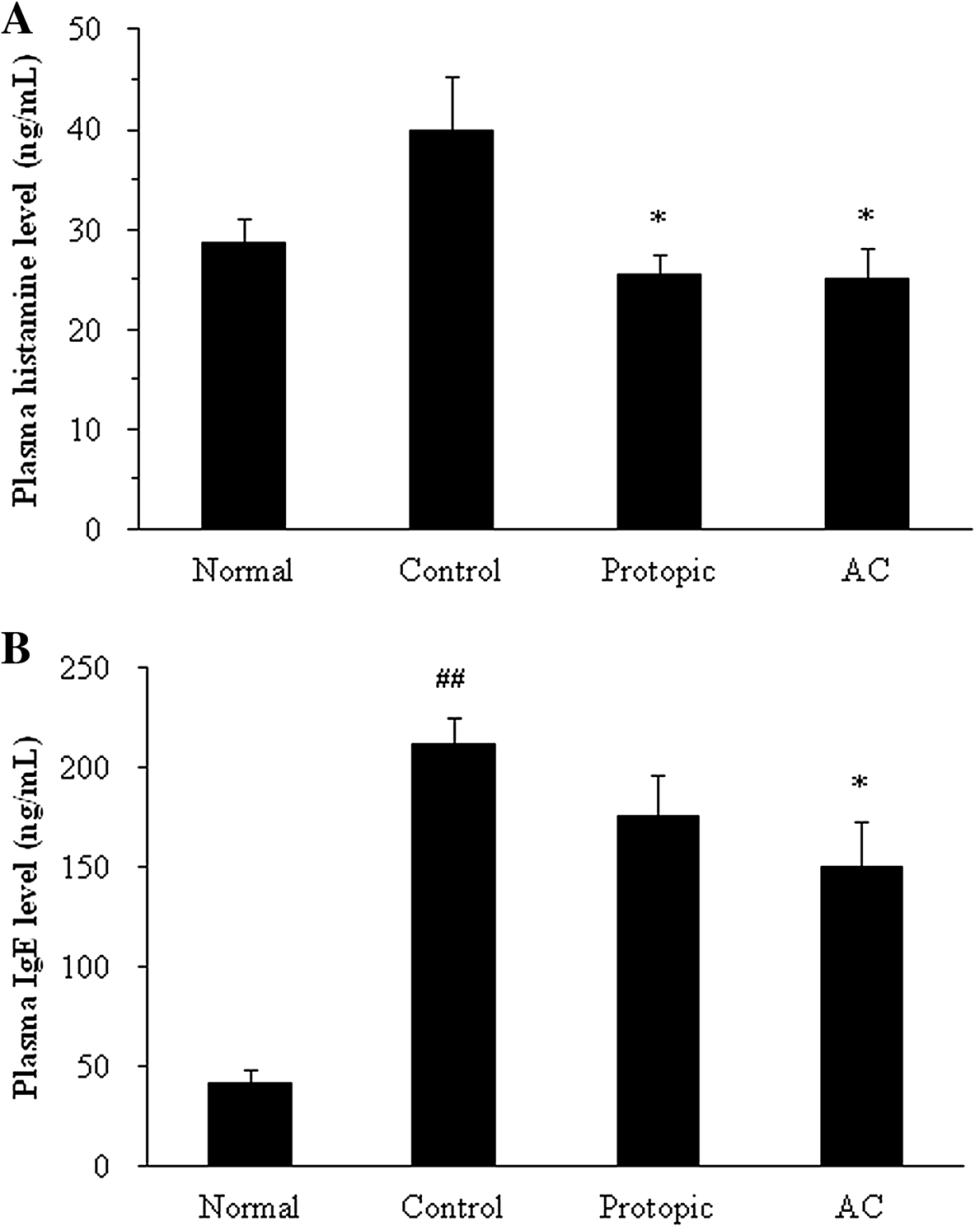
**The plasma levels of histamine (A) and IgE (B) in *D. farinae *extract-sensitized Nc/Nga mice.** AC (10 mg/mouse/day) and Protopic ointment (50 mg/mouse/day) were topically applied on the back and ears once daily for 4 weeks. The concentrations of histamine and IgE were measured by ELISA (mean *±* SEM, *n =* 5), ^##^: *P <* 0*.*01 compared with the normal group, *: *P <* 0*.*05 compared with the *D. farinae*-induced control group.

The plasma histamine levels in the control group (39.85 ± 5.44 ng/mL) were elevated compared to the normal group (28.56 ± 2.56 ng/mL). The histamine levels were reduced in the AC and Protopic treatment groups (25.12 ± 2.94 ng/mL and 25.54 ± 1.93 ng/mL, respectively) compared to the control group (*P* < 0.05, Figure 4A). Additionally, the total plasma IgE levels were significantly increased in the control group (211.3 ± 13.35 ng/mL) compared to the normal group (41.56 ± 7.022 ng/mL, *P* < 0.01). The treatment with AC inhibited increases in plasma IgE levels (149.9 ± 22.66 ng/mL) in *D. farinae*-sensitized mice (*P* < 0.01). However, there was no significant difference in the Protopic group (175.4 ± 20.36 ng/mL, Figure 4B).

## Discussion

AD is a common chronic cutaneous disease characterized by the over-expression of IL-10 and by high IgE levels. The most important allergens associated with human AD are house dust mite allergens, and *D. farinae* is the most common house dust mite present in the environment. The development of inflammation in AD is biphasic. An initial Th2 phase leads to a chronic phase associated with Th0 and Th1 cells [1]. The drugs currently used to treat AD are limited by the significant adverse effects associated with their long-term use. Recently, several studies have attempted to identify new candidates to treat AD with fewer side effects. Herbal remedies, including herbal medicines, are a popular trend in the field of complementary and alternative medicine. In previous reports, an extract of *A. capillaris* was shown to inhibit 5-lipoxigenase (5-LOX) activity in the RBL-1 cell line. The activity of 5-LOX is associated with several allergic and skin inflammatory disorders [11].

In the present study, the chemical contents of the AC extract were analyzed using a high performance liquid chromatography (HPLC) system. Kwon et al. reported that the major compounds of *A. capillaris* are scopoletin, scopolin, scoparone, esculetin, quercetin, capillarisin, isorhamnetin, 3-*O*-robinobioside, isorhamnetin 3-*O*-galactoside, and chlorogenic acid [10]. However, *Artemisia* species are very varied such as *A. capillaris*, *A. princeps*, *A. iwayomogi*, *A. ontana*, *A judaica*, etc. Moreover, it has been reported various compounds including coumarins (scoparone, scopoletin, scopolin, etc.), flavonoids (isorhamnetin, quercetin, isoquercitrin, hyperoside, etc.), chromones (capillarisin, 7-methylcapillarisin, etc.), phenylpropanoids (caffeic acid, chlorogenic acid, caffeoylquinic acids, etc.), lignans ((+)-sesamin, pluviatide, honokiol, etc.), and essential oils (*β*-pinene, *β*-caryophyllene, capillene, etc.) [13-19]. Among these different constituents, we were selected the six compounds including caffeic acid, chlorogenic acid, hyperoside, isoquercitrin, isochlorogenic acid A, and scoparone. Six compounds were selected as the marker compounds of *A. capillaris* in this study with references [13-15], and then performed simultaneous analysis using HPLC-PDA method. Isochlorogenic acid A and chlorogenic acid were detected as the major compounds in the AC extract. Isochlorogenic acid A was shown to have hepatoprotective effects and antioxidative properties through the induction of HO-1 [20]. Additionally, isochlorogenic acid A has immunopotentiation properties mediated through the NK-κB-induced release of NO from macrophages [21]. Chlorogenic acid has been reported to inhibit the production of inflammatory mediators and cytokines [22,23]. Moreover, chlorogenic acid exhibits antibacterial, antioxidant, anti-hepatic injury [24] and anti-allergic activities [25].

We investigated the anti-inflammatory and anti-AD effects of AC treatment using *in vitro* and *in vivo* systems because AD is strongly associated with the inflammatory response. Treatment with AC suppressed both the production of NO in LPS-stimulated RAW264.7 cells and the production of histamine in PMA/A23187-stimulated MC/9 mast cells. The involvement of Th2 cells both helps to explain the joint involvement of IgE-producing B cells (via IL-4 and IL-13), mast cells (via IL-4 and IL-10) and eosinophils (via IL-5) in the allergic inflammatory process and accounts for the other pathophysiologic features of allergy [26]. Nc/Nga mice are characterized by AD-like skin lesions and show elevated levels of blood IgE [27]. The infiltration of mast cells is another important factor in AD development. The dermatitis scores were reduced following AC treatment at the third week in *D. farinae*-sensitized Nc/Nga mice. In addition, AC suppressed the histological features of the disease, including edema, cornification of the epidermis, and mast cell infiltration in the dorsal skin and ear. Furthermore, treatment with AC reduced the plasma levels of histamine and IgE in *D. farinae*-sensitized Nc/Nga mice. As a result, topical application of AC reduced inflammatory response on destructive skin barrier functions, which is caused by allergens, such as *D. farinae*, in AD and thereby an increase of systemic IgE level was suppressed. IgE induces mast cell activation, which results in the release of preformed mediators, such as histamine, and cytokines (IL-4 and IL-13). It is expected that topical application of AC on the AD-like skin lesions reduces defective barrier functions by allergens and inhibits elevation of blood level of histamine via reduction of activated mast cells by reduced systemic IgE.

## Conclusions

Our results suggest that AC reduces the atopic dermatitis response of *D. farinae*-sensitized Nc/Nga mice via the inhibition of IgE-mediated mast cell degranulation. Additionally, our data indicate that there is a reduction in the release of preformed mediators, such as histamine. Therefore, we conclude that AC should be explored as a potential therapeutic agent to treat atopic dermatitis.

## Competing interests

The authors declare that they have no competing interests.

## Authors’ contributions

HH and HL designed the study and performed the experimental work and data analyses. CSS, HSL, JKL and MYL carried out the experiments. HH and CSS wrote the manuscript. HH and HS supervised the experimental work. All authors read and approved the final manuscript.

## Pre-publication history

The pre-publication history for this paper can be accessed here:

http://www.biomedcentral.com/1472-6882/14/100/prepub
